# Understanding the supports needed for policy implementation: a comparative analysis of the placement of intermediaries across three mental health systems

**DOI:** 10.1186/s12961-019-0479-1

**Published:** 2019-08-22

**Authors:** Heather L. Bullock, John N. Lavis

**Affiliations:** McMaster University, 1280 Main Street West, MML 417, Hamilton, ON L8S 4L6 Canada

**Keywords:** Intermediaries, implementation, mental health, behavioural health, healthcare, evidence-informed policy

## Abstract

**Background:**

Intermediaries are organisations or programmes that work between policy-makers and service providers to facilitate effective implementation of evidence-informed policies, programmes and practices. A number of intermediaries now exist in well-established mental health systems; however, research on them, and how they may be optimised to support implementation is lacking. This research seeks to understand the puzzling variation in the system placement of intermediaries supporting policy implementation in the mental health systems of Canada (Ontario), New Zealand and Scotland.

**Methods:**

Using a comparative case study approach, the analytic goal was to compare intermediaries across jurisdictions and explain differences in their placement using explanatory frameworks from political science. Data for this analysis were derived from several sources, including key informant interviews, a literature search of published and grey literature on intermediaries and on policy implementation in mental health systems, a review of relevant policy documents and websites, as well as documents and websites relating to the various intermediaries and other interest groups within each system.

**Results:**

Through the analysis, we argue that the placement of intermediaries supporting policy implementation can be explained through an understanding of the political structures, the policy legacies leading to the current public/private mix of mental health service delivery, and the differing administrative capacities of mental health systems.

**Conclusions:**

This research contributes to our growing understanding of policy-related intermediaries supporting implementation at scale and how we might build appropriate infrastructure in systems to support the implementation of policy and achieve better outcomes for citizens.

## Background

Governments are continually looking for better ways to achieve their policy goals. While policy implementation has been acknowledged as critical in filling the gap between policy promises and policy outcomes, the process itself is complex and multi-faceted and has yet to be well-understood. Policy implementation is generally defined as a series of activities undertaken by government and others to achieve the goals and objectives articulated in policy statements [1]. While governments and public administrators have clearly been interested in, and committed investments toward, implementation supports as part of the usual policy-making processes, most scholarly works have focused on designing policy to be ‘implementable’ or describing the factors that are important in the implementation process [2]. The literature is scant, however, when it comes to understanding how to build and harness the system resources required to support the policy implementation process [3]. Yet, there is growing recognition that the capacity of existing system actors (such as those who deliver health or social services to citizens) requires additional expertise and support to implement changes, especially those that are large-scale or complex in nature [4].

Thus, policy-makers and system leaders are increasingly turning to individuals (e.g. knowledge brokers or champions), teams (e.g. implementation teams), programmes or organisations with expertise in implementation science and quality improvement approaches to support such efforts. ‘Intermediaries’ are one such form of change agency.

### What are intermediaries?

In the most general sense, intermediaries are individuals, organisations or programmes that work in between existing system structures in order to facilitate communication or to achieve a particular goal. There are many different types of intermediaries that exist, playing a variety of roles in systems. One specific type of intermediary is those that are policy oriented and implementation focused. These intermediaries work in between policy-makers, on the one hand, and service providers, on the other, to facilitate effective implementation of evidence-informed policies, programmes or practices (EIPPs) through the use of specific implementation strategies [5, 6]. They play an important role as translators for policy and provide technical assistance (through the intentional application of specific implementation strategies such as assessing readiness, identifying barriers and facilitators to implementation, coaching and leading rapid improvement cycles, among others) to organisations and service providers that deliver services for citizens [7–10]. As the word suggests, intermediaries interface with a diverse array of organisations and coalitions that collectively comprise the ‘implementation infrastructure’ for a particular issue and are sometimes referred to as ‘backbone support’ in the collective impact literature [11]. Beyond direct service providers and policy-makers, these partners and stakeholders may include community coalitions, peak organisations, patient or citizen groups, professional bodies, researchers and research programmes, and unions, among others.

Most of the work done to date on intermediaries has been descriptive, often focused on their specific functions in systems. Scholars have identified many functions of intermediaries [12, 13] and some of the most commonly described functions include educating and stimulating interest in a policy or programme; assessing evidence and a policy or programme’s fit or feasibility in a certain context; linking knowledge generators and policy or programme developers with service deliverers; ensuring effective implementation and fidelity systems are developed and maintained; building capacity to implement well and integrate efforts to implement multiple initiatives; promoting the spread and scaling up of effective interventions; enabling quality improvement and quality assurance processes; and supporting policy and systems development.

Additionally, a recent study by Proctor et al. [14] found that intermediaries focused on the implementation of specific evidence-based practices for children and youth used an average of 32 discrete strategies with many of them focusing on planning, education and quality improvement.

The scholarship to date has been helpful in elucidating the important activities of intermediaries and how they can improve the capacity of service delivering organisations to implement changes. However, the intermediaries described in the literature do not always have a direct role supporting the implementation of policy. Instead, many are focused on supporting the implementation of one or more specific EIPPs at an organisational level. While the organisational level goals are generally not in conflict with the government policy directions, these efforts can, and often do, proceed without being tied specifically to the implementation of country or state/province-level policy direction. Yet, policy support is critical to scaling EIPPs across whole systems and for sustainment of implementation efforts over time. Furthermore, governments require implementation support to meet their own policy goals. There is therefore a need to further understand the role of policy-oriented, implementation-focused intermediaries as well as a need to go beyond description to offer explanatory accounts of such intermediaries in order to better understand them, with the longer-term goal of being able to use that understanding to optimise intermediary capacity to achieve better health and social outcomes for citizens.

### Implementation in mental health systems

In the policy domain of mental health, a focus on implementation is particularly important in order to achieve change because of the complex and multi-faceted nature of the system. What is loosely known as the ‘mental health system’ tends to be a suite of fragmented services delivered with varying levels of intensity and effect across services and sectors [3], making it challenging to achieve systemic change. A focus on implementation is also important because, while there is an increasing supply of evidence-informed treatments for a wide range of mental health and substance use problems, a number of studies have found that the majority of people experiencing such problems receive care that is not based on the best available evidence (e.g. [15–18]). Ensuring that mental health policy is evidence informed and facilitates the adoption of evidence-informed practices in service settings is critical to addressing this gap and reducing the unnecessary suffering of people with mental health and substance use problems.

Intermediaries can help to support the adoption of EIPPs in mental health systems and there are many examples of such intermediaries. For the purposes of this study we define policy-oriented, implementation-focused intermediaries (hereafter ‘intermediaries’) as organisations or programmes that have an explicit and recognised role to support the implementation of government mental health policy goals and employ specific methods of implementation support. These methods can range from quality improvement approaches to methods drawn from implementation science or knowledge translation. In order to achieve these goals, other actors in the policy system must understand and accept this role, including those in government. Intermediaries that focus on a single evidence-based programme or intervention, do not focus on a whole socio-political system (e.g. only work in particular communities within a socio-political system and do not operate at a province/state or national level), or that do not focus explicitly on the implementation of particular policy objectives related to mental health as their primary focus, are not included in the scope of this study.

### Study context

This research builds on an ongoing collaboration regarding implementation infrastructure in mental health systems that has been taking place from a group of countries that are part of the International Initiative for Mental Health Leadership (IIMHL) – an international collaborative that focuses on improving mental health services and systems in eight countries: Australia, Canada, England, New Zealand, Republic of Ireland, Scotland, Sweden and the United States (a ninth country, the Netherlands, has also recently joined). Beginning in 2013, a group of policy and system leaders from the IIMHL have been gathering virtually and in-person to consider and share lessons learned regarding the implementation infrastructure in their mental health systems and the role of intermediaries. One of the outputs of this collaboration was a descriptive summary report of a 2-day ‘intermediary organisation’ learning exchange held in Washington, DC, in 2016. Based on their shared experiences, the group noted that the intermediaries in their countries seemed to vary according to a number of structural and organisational dimensions despite playing a similar function in their systems. Most notable for the policy and system leaders was the variation in the placement of intermediaries in their systems. The lead researcher (H.B.) has participated in these meetings and the IIMHL became a partner on this research study. As partners, the IIMHL helped shape the research focus, provided endorsement and facilitated access to key informants, and became a receptive audience for, and provided feedback on, the findings.

Upon closer examination of the IIMHL-identified intermediaries as well as the published literature, our study team noted that the intermediaries seemed to vary in their placement in two key ways. First, there is a mix of the types of organisations that have assumed this function. In our examination of intermediaries that support mental health policy implementation, those uncovered thus far exist in six different system settings, namely (1) government (often as discrete programmes), (2) arms-length agencies of government (such as mental health commissions or quality agencies), (3) service delivery organisations, (4) non-governmental organisations (NGOs), (5) academic or research settings, and (6) ‘peak organisations’, defined as an organisation or association that represents a collective of similar organisations. Second, the intermediary function is often segmented in two different ways. Segmentation seems to be based on the age of the target population (child and youth versus adult) or by the sector (education versus health/mental health).

### Research question

Our research seeks to understand this puzzling variation in the system placement of intermediaries supporting mental health policy implementation. We ask the question – what influences how intermediaries are positioned in mental health systems? We hope that answering this question will contribute to our nascent understanding of the phenomenon of intermediaries and how we might build appropriate infrastructure in systems to support the implementation of policy and the achievement of policy goals.

## Methods

### Design

We used a comparative case study design [19] to explore the placement of intermediaries. To be congruent with our definition of an intermediary, each ‘case’ was defined as a socio-political system (either province/state or national) that had policy authority for mental health and the intermediary or intermediaries within.

### Sampling

We first looked for the presence of intermediaries in the mental health systems of the eight high-income countries that were part of the IIMHL at the time. Although England and Scotland are part of the United Kingdom, they are considered separate countries for this analysis because the governance authority for health and mental health rest with their respective National Health Services. They are all countries that have well-established health systems and their participation in the IIMHL reflects a commitment to mental health systems improvement and advancement. These countries provide adequate variation in terms of health service structures, including how mental health services are designed, managed and delivered. This sample pool also provides adequate variation in the factors that may impact successful implementation but enough similarity in the underlying features of the systems (government spending per capita, etc.) to ensure the analysis is sensitive to the variables of interest.

### Case selection and justification

The criteria used to select cases for this analysis included (1) the presence of an intermediary that met our definition; (2) the intermediary(ies) was well-established with multiple data sources from which to draw; and (3) there was variation in the dependent variable (the system placement of the intermediary). We aimed for three cases to keep the comparison manageable. Based on these criteria we purposively sampled the jurisdictions of New Zealand, Canada (Ontario) and Scotland for this analysis. New Zealand is a unitary state and authority and policy decision-making for healthcare and mental health rests nationally and the intermediary function is also a national body. In Canada, Ontario was selected because it was the province with the most well-developed intermediary structure aligning with our definition, and despite it not being a national example, due to Canada’s federalist structure, healthcare (mental health) is primarily under the jurisdiction of the provinces. Scotland was included because it had the most well-developed intermediary structure located in government and there were many publicly available data sources from which to draw. By selecting jurisdictions that share similar macro-system features, we reduce the possibility that variation in these features alone can be the explanation for why there is variation in the placement of intermediaries.

### Data collection

#### Qualitative interviews

One or two leaders from each jurisdiction were invited to participate in a brief phone interview with the study team. The questions focused on four areas – (1) structures supporting implementation of mental health priorities (where implementation functions exist within their system, who is responsible for carrying out implementation and what skills they have); (2) methods for change being utilised (such as quality improvement, implementation science, etc.); (3) how established these structures and methods are and whether they have evidence of their effectiveness; and (4) health system characteristics (to provide an overview of the key features of the mental health system in terms of governance, financial and delivery, such as mental health priorities currently identified, dedicated funding, etc.) and political system characteristics (such as institutional arrangements, interest group dynamics, dominant values, etc.). Key informants were asked for any supporting documents or websites that describe their system’s characteristics or implementation structures or methods in detail. In total, nine interviews were conducted, with participation from all countries except for Scotland. Despite Scottish leaders not being able to participate formally during the study period, one of the researchers (H.B.) had informal conversations and heard formal presentations from members of the Scottish intermediary structure through IIMHL activities just prior to the study period. A mix of leaders participated, including those in government, agencies of government, NGOs and service providers who had roles related to implementation.

Interviews varied in length from 30 min to 1 hour. Interviews were conducted via Skype (voice only) and were recorded with the permission of the key informant using a recording software programme. Written notes were also taken by the interviewer. Summary sheets were created after each call with sections mapping to the four areas of interest (above).

#### Document review

We reviewed several documentary sources, including (1) a literature search of published and grey literature on intermediaries using the terms “intermediar*”, “intermediary organi*”, “knowledge brokering organi*” and “backbone organization” using PubMed and PsycInfo; (2) a literature search of published and grey literature on policy implementation in mental health systems using PubMed and PsycInfo; (3) a review of policy documents (including presentations) and government websites, including current and past mental health strategies, targets and indicators, and background documents pertaining to their development; and (4) a review of documents and websites relating to the various intermediaries and other interest groups within each system.

### Analysis

First, data relating to the placement of intermediaries, their role in systems, and the methods they use from the interview transcripts and documents were extracted using qualitative description [20]. Next, all data sources were analysed again with an explanatory lens using directed content analysis [21], whereby the analysis is guided by existing theory. Institutional theory (and historical institutionalism specifically) was used to explain the observed differences in placement. The focus at this stage was comparing across jurisdictions.

### Ethical considerations

Ethics approval for this research was granted by the Hamilton Integrated Research Ethics Board (HiREB Project #15–328).

## Results

### I – Description of the intermediaries (and their system placement)

Table 1 provides a summary description of the intermediaries in each jurisdiction. It is important to note that some of the functions ascribed to intermediaries (e.g. [13]) may exist in other parts of the mental health systems of these jurisdictions. Those described below and used in the analysis are those that fit the definition most clearly and are the most recognisable form of intermediary from a mental health policy implementation perspective.

**Table 1:** Intermediary structures in New Zealand, Ontario and Scotland

Intermediary name	Placement in system	Year est.	Number of staff	Approx. annual budget	Funding source	Location(s)	Structure	Description	Implementation methods
New Zealand									
Te Pou o Te Whakaaru Nui (Te Pou)	Non-governmental organisation (NGO)	2006	46	$20 million NZD	Ministry of Health (primary source)	• Auckland • Hamilton • Wellington • Christchurch Travel as needed across the country	• Part of Wise Group of community organisations Governance: • Board of directors (7 members) • Clinical sector reference group (26 members) provides advice to ensure clinical and sector partnership feedback is incorporated • 1 of 5 national workforce development centres for mental health	• National centre of evidence-based workforce development for the mental health, addiction and disability sectors • Works with range of organisations and people, including service providers (District Health Boards and NGOs), training and education providers, researchers and international experts, to educate the workforce and support improvements to services	• Knowledge exchange (e.g. SPARK Evidence into Practice) • Decision support/data systems (collecting and reporting on outcomes and workforce information) • Capacity building of system to access, interpret and implement evidence • Technical assistance (e.g. seclusion and restraint reduction) • Strong focus on collaboration
Ontario, Canada									
Ontario Centre of Excellence for Child and Youth Mental Health (OCoE CYMH) Children’s Hospital of Eastern Ontario (CHEO)	Service delivery system - hospital	2004	50	$5.9 million CAD	Ministry of Child & Youth Services	• Ottawa Travel as needed across province	Governance: • CHEO hospital board • Strategic advisory council (12 members) provides advice, direction and input on strategic plans, partnership initiatives and high-level operations	• Focus on increasing capacity in the child and youth mental health service delivery system to use evidence-based practices, evaluate their work, and to improve their ability to collaborate across systems with the goal of improving services	• Implementation science approach (e.g. National Implementation Research Network’s (NIRN) Active Implementation Frameworks) • Knowledge mobilisation • Quality improvement • Performance measurement • Evaluation
Provincial System Support Program Centre for Addiction and Mental Health (CAMH)	Service delivery system - hospital	2011	150	$19 million CAD	Ministry of Health and Long-Term Care and global hospital budget	Provincial office: • Toronto Regional offices: • Kenora • Thunder Bay • Sudbury • Barrie • London • Hamilton • Ottawa • Kingston • Toronto	Governance: • CAMH hospital board	• Works collaboratively across sectors to move evidence to action to transform mental health and addictions systems in Ontario	• Implementation science (e.g. NIRN’s Active Implementation Frameworks) • Knowledge exchange • Information management • Evaluation • Engagement and health equity
School Mental Health Assist (SMH Assist) Hamilton-Wentworth District School Board	Service delivery system – school board	2011	13 provincial staff, 72 mental health leaders in schools	$2.2 million CAD (figure does not include funding for mental health leaders)	Ministry of Education	Provincial office: • Hamilton Regional offices: • In all 72 school boards	Governance: • Hamilton Wentworth District School Board (Director of Education)	• Created to address critical gaps in the organisational capacity and conditions of schools and school boards to provide evidence-informed programming addressing mental health (Short 2016)	• Implementation science approach, drawing from NIRN’s Active Implementation Frameworks
Scotland									
Quality and Efficiency Support Team and Mental Health Division of Scottish Government	Government	2009 (restructured in 2012)	12	N/A	Scottish Government	• Edinburgh Travel as needed across country	Governance: • Mental Health Delivery Team (12 people drawn from multiple agencies) • Scottish government	• Focuses on elements of a quality improvement and outcomes framework • Ensures the delivery of commitments relating to measurement of progress and improvement support	• Quality improvement - adapted version of Institute for Healthcare Improvement’s Model for Improvement [34] • Monitoring of progress toward Health improvement, Efficiency, Access to treatment, Treatment targets

#### New Zealand (NGO)

Te Pou o te Whakaaru Nui (Te Pou) is a NGO that acts as a national workforce development centre for mental health, addictions and the disability sector in New Zealand. Established in 2006, it is part of a larger group of community organisations under the umbrella of the Wise Group (www.wisegroup.co.nz) and receives funding through the Ministry of Health in New Zealand. Te Pou’s role is to enhance practice development; ensure services are informed by the best available evidence; support the collection and use of outcomes and workforce information; and inform future policy [22]. Along with workforce development being identified as a priority for policy and thus supporting the implementation of this policy direction (e.g. Rising to the Challenge: Mental Health and Addiction Service Development Plan, 2012–2017 [23]), Te Pou also supports the implementation of other policy priorities such as those related to primary care, the peer workforce, and reducing the use of seclusion and restraints. They have staff in several locations across the country, increasing their ability to support different regions. Their focus on ensuring evidence informs practice has meant an increasing reliance over time on knowledge exchange and implementation science tools and frameworks as well as promoting the capacity of others in the mental health system to use, access, interpret and implement evidence in their settings (e.g. SPARK: Evidence into Practice).

#### Ontario, Canada (service delivering organisation)

In Ontario, three intermediaries have emerged to support the policy directions of the provincial government. All three exist within the service delivery system – two through hospitals and one through a school board. In 2011, a new programme was established at the Centre for Addiction and Mental Health, Canada’s largest psychiatric academic health sciences centre. The Provincial Systems Support Program was created in response to Government of Ontario’s request of the Centre for Addiction and Mental Health to lead several key provincial activities. Capitalising on existing system capacities, the programme developed expertise in five key areas, namely knowledge exchange, information management, implementation, evaluation, and engagement and health equity [24]. One of the larger implementation efforts has been in support of one of the major activities identified in Open Minds, Healthy Minds Ontario’s Comprehensive Mental Health and Addictions Strategy: “*Create 18 service collaboratives to support coordinated services for children, youth and adults, including a focus on children and youth in transition from inpatient to outpatient settings, between health and justice systems, and child-focused to adult services*” ([25], p. 23) with the support of six Ontario government ministries. The Provincial Systems Support Program draws from a range of different frameworks to support its implementation work, including the National Implementation Research Network’s (NIRN) Active Implementation Frameworks [26, 27].

Another hospital-based intermediary in Ontario is the Ontario Centre of Excellence for Child and Youth (OCoECYMH). In 2004, the then newly established Ministry of Child and Youth Services provided funds to the Children’s Hospital of Eastern Ontario to establish the OCoECYMH [28]. The original objectives of OCoECYMH focused on increasing capacity in the child and youth mental health service delivery system to use evidence-based practices, to evaluate their work and to improve their ability to collaborate across systems with the goal of improving services for children and youth in Ontario [28]. Over time, their work has become increasingly focused on supporting agencies to successfully implement changes, as well as supporting the implementation of the policy directions of the Ministry of Child and Youth Services. Most recently, they have begun to support the implementation of the system transition efforts underway related to Moving on Mental Health – A System that Makes Sense for Children and Youth [29]. In order to achieve these goals, they draw from a number of theories and frameworks related to knowledge translation, knowledge exchange and implementation science that they have tailored to fit their context.

School Mental Health Assist (SMH ASSIST) is a third intermediary that is playing an active role supporting the implementation of the Ontario government’s policy directions. It is based out of the Hamilton-Wentworth District School Board. SMH ASSIST was created in 2011 to address critical gaps in the organisational capacity and conditions of schools and school boards throughout the province to provide evidence-informed programming addressing mental health [30]. SMH ASSIST received funding directly related to Open Minds, Healthy Minds (2011) through the Ministry of Education. This funding was directed through the Hamilton-Wentworth District School Board on behalf of the initiative. SMH ASSIST includes an implementation team that works across the province to support newly funded mental health leaders linked to each school board in the province that were supported as part of the Open Minds, Health Minds commitment. SMH ASSIST draws heavily on the National Implementation Research Network Active Implementation Frameworks model as their approach to implementation.

#### Scotland (government)

After seeing limited ability of local health boards to embed and sustain the changes outlined in several key strategic policy documents (e.g. Delivering for Mental Health [31]; Towards a Mentally Flourishing Scotland [32]), the Scottish government created several focused elements of a quality improvement and outcomes framework. These included (1) the development of a mental health quality and outcomes framework; (2) specific Health improvement, Efficiency, Access, Treatment targets that are tied to the performance of chief executives in the health boards; (3) benchmarking key indicators and the development of a balanced scorecard for health boards to achieve; and (4) the development of a mental health collaborative with funds to support the capacity of health boards to improve. To oversee this work, the Scottish government’s Quality and Efficiency Support Team combined efforts with the Mental Health Division of Scottish Government. With the more recent national Mental Health Strategy for Scotland 2012–2015 [33], the country also developed a Mental Health Delivery Team responsible for overseeing its progress, including monitoring the performance of NHS Boards against the mental health Health improvement, Efficiency, Access, Treatment targets. Comprised of individuals from the key national bodies with responsibility for mental health, the team has specific responsibility to ensure delivery of commitments relating to measurement of progress and improvement support as well as those commitments that do not currently sit within the framework of the other implementation and monitoring groups in the country. The approach to implementation by the Scottish government draws from the Institute for Healthcare Improvement’s Model for Improvement (http://www.ihi.org/resources/Pages/HowtoImprove/default.aspx), which has been adapted according to the goals of the work and the Scottish context and is outlined by Coia and Glassborow [34].

What is interesting about these three jurisdictions is that their intermediaries are not new organisational forms. Rather, the function of policy implementation support has been built into existing institutional infrastructure. This can be best described as a process of institutional conversion, which, according to Thelen [35], occurs when “*existing institutions are redirected to new purposes, driving changes in the role they perform and/or functions they serve*”. In these cases, the conversion process is only partial in nature, since all of the initial institutional functions continue to be filled. For example, the NGO in New Zealand continues to do the workforce development work it was originally established to do, the hospitals in Ontario still continue to serve patients and the government in Scotland still fulfils its other governmental duties. The conversion in this case consists of scaffolding a new function onto an existing organisation rather than replacing the function outright.

It is also important to note that, in each of these jurisdictions, some of the intermediary functions are fulfilled by other organisations or programmes in other locations in the mental health systems. The intermediary role tends to be distributed across systems with different organisations contributing different types of expertise and fulfilling different but complementary functions. The focus here is on those that have the most direct and concentrated functions.

### II – What explains the placement of intermediaries in these mental health systems

We propose that the system placement of intermediaries in these three mental health systems can be explained primarily by drawing on historical institutionalism. Health systems in general, and mental health systems in particular, need to adjust their policy implementation strategies to fit the contours of different institutional terrains. It is these differing institutional landscapes that explain the variation in system placement of the intermediaries. Our analysis indicates that two factors in particular, namely the variation in public–private mix of mental health service delivery due to legacies from past policies and the differing administrative capacities of mental health systems, collectively explain the differences in where intermediaries are located.

#### Policy legacies leading to a differing public–private mix of health and mental health service delivery

The mix of health and mental health services that are publicly delivered compared with those that are delivered by private for-profit or not-for-profit entities (hereafter public–private mix) due to policy legacies helps to shed some light on when we might see intermediaries within government. Intermediaries are more likely to be part of government in jurisdictions that have public delivery of mental health services with no alternative service streams, but a public–private mix alone does not explain where intermediaries might be located if this is not the case. However, it does suggest that leading policy implementation may be a valuable function for other systems actors to take on since it could reinforce their role in the system and provide them with access to elites and additional financial resources.

The public–private mix of health and mental health service delivery creates different incentives or disincentives for system actors. Actively leading the implementation of policy is visible and traceable to a wide range of system actors and also to the public, depending on the specific direction being implemented [36]. The visibility and traceability of policies and policy actions (including implementation) can convey important information to system actors that can influence their attitude and behaviours [37]. While implementation success can lead to concentrated gains for those leading it, implementation failure is easily traced back to the leaders, causing concentrated losses. For the government, or any other actor, being actively engaged in the implementation of mental health policy is risky. Because of the complexity of the problem, the policy solutions are often complicated and multi-faceted, spanning a wide array of system actors and different sectors. This increases the likelihood that implementation efforts may not achieve the intended results or results may take longer than in some other policy arenas. For governments that act as what Weaver [38] calls “*blame avoiders*”, this may mean it is advantageous to “*pass the buck*” of mental health policy implementation to other system actors when feasible. Conversely, while other institutional actors also face the risks related to implementation, they do not face the same losses as government, such as losing the ability to govern through the electoral process. By leading the implementation of the government’s policy directions, system actors can receive other benefits, such as increased access to government elites and financial resources, which secure or even increase the centrality of their place in the system.

In all three jurisdictions funds are raised for healthcare primarily through taxation and all have some form of universal insurance for citizens. Where they differ is primarily in the delivery of services and this difference, we argue, is important in explaining the placement of intermediaries to support implementation. When governments are directly responsible for the public delivery of services (as is the case in Scotland through the Scottish National Health Service) they are viewed by themselves and other system actors as having certain powers and authorities that would not be attributed to governments in other systems where service delivery is more ‘divorced’ from government. Scotland’s mental health system constitutes the most centralised and government-concentrated form of service delivery of the three cases. In such a system, one might expect that the government could also be directly involved in the implementation of new policies because assuming the role of intermediary would be viewed as a logical role for them. They also have less opportunity for blame avoidance [38] because there are fewer institutions in the mental health service delivery system to which they can shift responsibility.

Conversely, in Ontario, mental health delivery is more arms-length from government, provided mainly through private, not-for-profit community mental health agencies and hospitals. However, unlike most other areas of healthcare, there is also a goodly amount of private, for-profit delivery, including registered professionals such as psychologists and social workers operating in private practice and some private mental health and addiction residential treatment facilities. In this type of service delivery environment, many of the specific service delivery decisions are made by governing boards that follow the broad policy expectations and service contracts outlined by the provincial government and the regional health authority structure (Local Health Integration Networks). Implementation support related to new policy directions is not under the auspices of the government, which sees itself as a ‘steward’ of the health system and less involved with the actual delivery of health (and mental health) services.

Somewhere in between these two cases in terms of public–private mix is New Zealand. The mental health service delivery system in New Zealand includes a mix of public, private and NGO providers. The Ministry of Health flows funds to 20 District Health Boards (DHBs) that are responsible for providing some portion of services directly. DHBs also purchase services offered by NGOs, primary healthcare organisations or other private providers. NGOs account for approximately 30% of the mental health and addiction service delivery budget of DHBs [22]. Citizens are also able to pay for private services directly, either out-of-pocket or through additional private insurance. This system might include more government capacity than Ontario to directly support the implementation of policy directions, since it includes some portion of direct government delivery. However, its mixed model of service delivery from a range of provider types means that it is not necessarily an obvious place for an intermediary. Unlike Scotland, in both New Zealand and Ontario, there are other institutional actors that could be tasked with supporting policy implementation because past policy decisions have led to less centralised forms of mental health service delivery. These capacities would allow blame-avoiding governments to ‘pass the buck’ to other system actors, who could take on this risky role. Furthermore, as mentioned, these other system actors operate with a different mix of incentives and are not subject to some of the concentrated costs that implementation failure brings government, making this role more palatable and, in fact, potentially desirable.

In order to explain which system actors might assume this role, we must turn to another institutional feature of mental health systems.

#### Administrative capacities

Governments vary in the degree to which they possess the resources needed to implement policies and decision-makers must consider not just the political constraints related to a given policy, but also the administrative and financial ones [39]. We suggest that intermediaries are created in the system location that has the most administrative capacity to enact the functions required of them and that this capacity was built as a result of past policy decisions. Administrative capacities can be broken down into two sub-categories, as follows: (1) human resource capacity, or what Pierson [39] called “*loyal and skilled*” staff, and (2) functional capacity, which refers to the practical ability of the system to support the intermediary function through the efficient flow of funds and other resources. Each of the jurisdictions examined here have their own particular history, replete with past policy decisions that over time build and shape each system in a unique way. As Skocpol states “*Because of the official efforts made to implement new policies using new or existing administrative arrangements, policies transform or expand the capacities of the state. They therefore change the administrative possibilities for official initiatives in the future, and affect later prospects for policy implementation*” ([40], p. 58).

Implementation support delivered through intermediaries requires very skilled individuals that are able to work ‘in between’ and understand both government and service delivery environments. They must also offer expertise in one or all of quality improvement, implementation science or knowledge translation. Finally, they must be skilled communicators who are able to translate policy intention into change at a service level. This is similar to the role of policy entrepreneur described by Kingdon [41]. Whereas policy entrepreneurs play a crucial function in coupling the problems, politics and policy streams to bring an issue to the decision agenda, those working as intermediaries play a crucial role in facilitating implementation by working effectively with actors at the policy, managerial and front-line levels. Each jurisdiction will vary in terms of where such human resources are found or where this capacity can be built.

Functional capacity, on the other hand, is the capacity built from previous policy decisions around how funds can flow through the mental health system and to whom. Although a key function of government is to flow funds to other actors in their system, at any given time, governments are constrained in their ability to disperse resources to certain actors with whom they have no prior existing financial relationship. It is always easier and swifter to use existing administrative capacity that exists from past policies, allowing funds to flow relatively rapidly and with little question about why from other system actors than to construct new financial arrangements. Governments then, have an important incentive to continue to use these pre-existing pathways to achieve new policy implementation support.

##### New Zealand

The New Zealand government works closely with NGOs that receive ‘significant funding’ on the scale of NZD$2–4 billion per year for health, with funding to NGOs for mental health and addictions representing approximately one-third of the total budget [42]. The government also recently formalised this relationship with NGOs through the development of a Health and Disability NGO council and Network. This partnership supplements the government’s capacity to provide mental health and addiction services and supports, but NGOs also play a key role in systems support, including workforce development, anti-stigma initiatives, and making service information and resources available for self-support [42]. The NGO sector represents significant human resource capacity, with a skilled workforce constituting a diverse range of roles. Te Pou in particular has the type of human resources articulated above that are able to fulfil the intermediary functions. Furthermore, Te Pou’s presence across the country and their existing relationships with the wide array of organisations delivering services means they have the functional capacity to play this role. Thus, the administrative capacity of the NGO sector, in general, combined with the specific human resource and functional capacity of Te Pou makes it a logical place for the intermediary function in New Zealand.

##### Ontario

The Ontario government adopted a stewardship model of governing in health in 2007, where it shifted its focus to providing overall direction and driving strategy and performance and became less directly involved in the actual delivery of healthcare. It also devolved some decision-making authority to the newly created regional health authorities (Local Health Integration Networks), which have been mainly focused on service contracting. These changes have meant there is limited administrative capacity within government to support an intermediary function. While there are many interest groups within the mental health sector, including a number of NGOs, they are very limited in size and scale and tend either to play an advocacy role or are association driven, representing the interests of service-providing organisations and providing them with group insurance and other benefits. Although some of these organisations receive funding from government to support specific activities, and thus have the functional capacity to receive funds from government to play an intermediary function, they tend not to have the mix of human resources with the right skills and supports to make them a logical site for an intermediary. Alternatively, the institutional service delivery sector in Ontario is robust and both hospitals and school boards are well recognised and trusted by government. They are also large in size and have a well-trained, highly skilled workforce. Additionally, these institutions have traditionally engaged in many activities that go beyond service delivery such as research, community development and continuing education. Furthermore, the functional capacity exists for government to flow funds to these organisations directly. Logic then dictates that the system actors who would receive funds to develop the policy implementation support function in the form of an intermediary would be service-delivering organisations.

##### Scotland

As mentioned, Scotland’s mental health system constitutes the most centralised and government-concentrated form of service delivery of the three cases. The Scottish Government’s direct involvement in the implementation of new policies is aided by their existing administrative capacity related to the delivery of services in the system, including a bureaucratic workforce with a diverse range of administrative skills and expertise from which to draw [36]. Additionally, as part of a larger governmental thrust, Scotland has reshaped its mental health system around a focus on improvement through the creation of specific mental health improvement aims, targets and improvement supports, and more generally through the establishment of Health Improvement Scotland in 2011. Health Improvement Scotland represents a functional system capacity that can support the skilled members of the government workforce who work closely with the service delivery system by enhancing their expertise in improvement approaches. These administrative capacities combine to reinforce the intermediary function played by the current mental health delivery team within government.

## Discussion

This analysis demonstrates that the system placement of intermediaries in these three jurisdictions is explained by their institutional landscapes and in particular, the mix of public–private mental health service delivery created by policy legacies and the differing administrative capacities of their systems. A system such as Scotland, with public delivery and administrative capacity within the government, is more likely to have the intermediary function within that setting. When delivery is a public–private mix (like New Zealand) or primarily private (like Ontario), then the location of the intermediary is explained by where the administrative capacity exists in the system (NGO sector in New Zealand and service delivery system in Ontario).

A key strength of this study is that it is an early attempt to combine theory on facilitation from implementation science with theories from public policy and other social sciences to explain intermediaries supporting mental health policy implementation. By drawing on historical institutionalism theory, it offers an explanation for the placement of intermediaries in systems. This study also provides rich descriptions of intermediaries in three different jurisdictions – something that is currently lacking in the literature – and an important building block to clarifying the phenomenon of intermediaries.

A limitation of this analysis is the lack of interview data from Scotland. It is possible that key informant interviews from that jurisdiction may have altered or served to enrich the analysis and conclusions that were drawn. However, the research team was in contact with key leads in Scotland just prior to the study period and was able to draw on presentation and other publicly available materials to mitigate this limitation. Additionally, this analysis did not include cases of intermediaries from the other three settings identified (arms-length agencies of government, academic or research settings, or peak organisations). Intermediaries do exist in these settings and their inclusion could further test the institutional arguments forwarded here and increase the conceptual credibility of the conclusions. Furthermore, it is possible that locations of intermediaries were overlooked or misclassified. For example, research in the field of education examining ‘knowledge mobilisation intermediaries’ identified four possible types – government, not-for-profit, for-profit and membership [43]. This classification diverges from that used here and identifies two potential other categories for system placement, namely for-profit and membership settings.

This analysis yields a set of testable hypotheses that can be used to examine the emergence and system placement of intermediaries in other mental health systems or other areas of health or social care. In particular, it would be interesting to compare jurisdictions where intermediaries exist with jurisdictions where they do not to explore what system features explain how and why they come about. Future research could also investigate whether the system location of intermediaries in systems affects the type activities they engage in or the relative weight of activities. One might predict that intermediaries in the delivery system would have a strong organisational-level focus and an emphasis on organisational-level activities. Those within government may be more focused on the implementation of policy goals and targets. Intermediaries in academic settings might have an increased focus on the purveyance of evidence-based practices or the translation of research evidence for policy and practice.

## Conclusions

A better understanding of intermediaries is important for policy-makers who must consider the infrastructure required to support the implementation of policy. This study offers them insights about where they might build such capacity and the types of intermediaries that are possible. It represents a unique contribution to the growing literature on intermediaries, but more work is required to truly understand how to harness systems more effectively to achieve policy goals.

## Data Availability

The data that support the findings of this study are available on request from the corresponding author [HB]. Some data are not publicly available due to them containing information that could compromise research participant privacy/consent.

## Abbreviations

DHBs: District Health Boards
EIPPs: evidence-informed policies and practices
IIMHL: International Initiative for Mental Health Leadership
NGO: non-governmental organisation
OCoECYMH: Ontario Centre of Excellence for Child and Youth Mental Health
SMH ASSIST: School Mental Health Assist
Te Pou: Te Pou o Te Whakaaru Nui
